# Total Tumor Irradiation for Multiple Lung Metastases Using Carbon Ion Radiotherapy and High-Frequency Oscillatory Ventilation: A Case Report of Two Patients

**DOI:** 10.7759/cureus.79069

**Published:** 2025-02-15

**Authors:** Yan-Shan Zhang, Yi-He Zhang, Yee-Min Jen, Yi Wang, Xiao-Jun Li

**Affiliations:** 1 Department of Radiation Oncology, Heavy Ion Center, Wuwei Cancer Hospital, Wuwei, CHN; 2 Department of Radiation Oncology, Yee Zen General Hospital, Taoyuan, TWN; 3 Department of Intensive Care Unit and Anesthesiology, Heavy Ion Center, Wuwei Cancer Hospital, Wuwei, CHN

**Keywords:** 15 or more metastatic lesions treated by radiotherapy, carbon ion radiotherapy, case report, high-frequency oscillatory ventilation, multiple lung metastases, novel local treatment for polymetastases, total tumor irradiation

## Abstract

Curative-intent radiotherapy for multiple lung metastases with more than 10 lesions is limited by lung dose constraints and respiratory motion. Carbon ion radiotherapy (CIRT) leverages the Bragg peak for precision and hypofractionation, while high-frequency oscillatory ventilation (HFOV) minimizes motion under anesthesia. This study evaluates the feasibility of combining CIRT and HFOV in treating ≥15 lung metastases. Two patients received single-fraction CIRT (50 Gy(RBE)) targeting all lesions. Case 1 (16 metastases of bilateral lung, hepatocellular carcinoma) and Case 2 (15 metastases of the left lung, colorectal adenocarcinoma) underwent four-dimensional (4D)-CT simulation with HFOV and CIRT in one single dose. Diaphragm motion reduced from 1.15 and 3.09 cm for Cases 1 and 2 (free-breathing) to 0.1 and 0.2 cm, respectively. Lung dose constraint parameters (V30: 3.57% to 12.35%; V20: 6.06% to 15.3%) met safety thresholds. Both patients achieved 22-month survival with continued local control (complete response in Case 1; partial response in Case 2). Case 1 developed grade II pneumonitis (resolved with steroids) and asymptomatic fibrosis; Case 2 had no toxicity. Some new lesions were managed with additional CIRT. Outcomes compare favorably to published photon trials in which up to 10 lung lesions were irradiated, underscoring CIRT’s precision and HFOV’s motion control. Success relied on three factors: CIRT’s dosimetric advantages, HFOV-driven motion reduction, and anesthesia-enabled tolerability of the longer irradiation time span. Despite cost barriers, CIRT seems to extend survival in patients with multiple lung metastases that were refractory to conventional local therapy. This approach demonstrates the feasibility of irradiating more than 15 lesions in patients with polymetastatic lung disease, offering durable control with manageable toxicity. Larger studies are needed to validate its role in multimodal therapy.

## Introduction

While irradiating all the metastatic lesions in a patient with oligometastasis with curative-intent radiotherapy is becoming popular, treating all metastatic sites with definitive radiotherapy in a patient with 10 or more polymetastases diffusely scattered in the lung is not yet the standard of care. Safety is a major concern because the dose constraint of the normal lung will be exceeded when multiple lesions are irradiated. However, the boundary between oligo- and polymetastases is not uniformly defined, ranging from three to 10 lesions for oligometastasis. In a joint consensus from Europe and the US, the maximum number is no longer used to define the status of oligometastasis; instead, the possibility to safely deliver curative intent metastasis-directed radiotherapy determines the status of oligometastasis, reflecting the pivotal role of safety in irradiating multiple lesions [1].

Apart from safety, the second issue is organ motion that could reach 5.6 cm in the cephalic-caudal direction [2]. Even though these two obstacles are overcome, local radiotherapy to so many lesions with any existing motion control technique would be impossible considering the protracted radiation time.

Carbon ion radiotherapy (CIRT) is an emerging new precision radiotherapy technique. Compared to photon radiotherapy, CIRT has the physical advantage of a Bragg peak and several unique biological advantages [3-7]. The Bragg peak of carbon ion gives CIRT the unique feature of a minimum dose after it, resulting in a much better normal tissue sparing compared to photon radiotherapy. Clinically, CIRT is given with fewer fraction numbers than photon radiotherapy.

Ventilator-assisted respiration motion control with various types of high-frequency ventilators has been reported in the literature using high-frequency jet ventilation (HFJV), high-frequency percussion ventilation (HFPV), and continuous positive airway pressure (CPAP) ventilation [8-11]. High-frequency oscillatory ventilation (HFOV) is a technique commonly used in the protection of lungs for patients with acute lung disease. By using a very high respiratory frequency and a low tidal volume, HFOV allows gas exchange, maintains a constant mean airway pressure, and, in the context of radiotherapy, minimizes respiratory movements [12]. Compared to HFJV and HFPV, HFOV has the advantage of minimal pressure lung damage. There are no reports using HFOV as an organ motion control method in the literature.

We report on two patients who received a single high dose of carbon ion to 15 and 16 lung lesions, respectively, with prolonged local tumor control and no high-grade toxicities. The purpose is to demonstrate the feasibility of definitive radiotherapy directed at 15 or more lung metastatic lesions in selected cases, specifically by applying CIRT and motion control with HFOV.

## Case presentation

**Figure 1:**
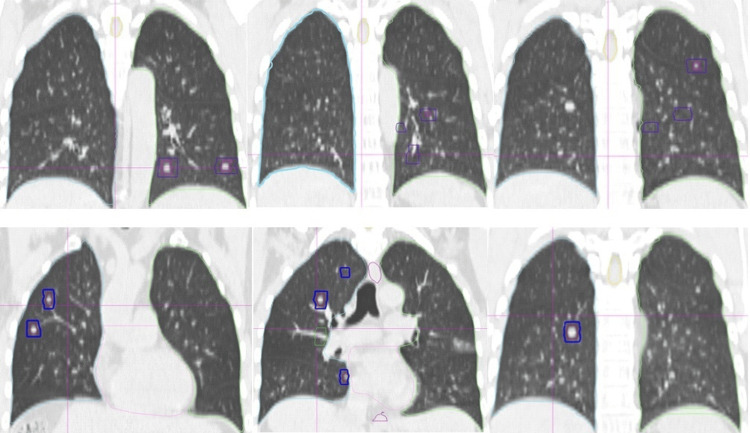
Target delineation of total lung carbon ion radiotherapy (CIRT) of Case 1 presented in coronal CT images with the lung window. Because the lesions were spread three-dimensionally, not all the 16 targets are visible in the figures. The targets of the left lung (upper panel) and right lung (lower panel) are shown separately. Red: gross target volume (GTV); blue: planning target volume (PTV).

Case 1 was a 51-year-old male patient with hepatocellular carcinoma and liver cirrhosis after resection of the segment 5. The stage was pT1bN0M0, stage IA by the American Joint Committee on Cancer (AJCC) criteria, 8^th^ edition. He had the comorbidity of hepatitis B carrier status and type II diabetes. Multiple lung and bone metastases were detected two months later. After salvage bevacizumab and sintilimab, the disease progressed with an increased number of lung lesions. The diagnosis was Barcelona Clinic Liver Cancer (BCLC) stage C. On March 8, 2023, all of the 16 lung lesions were irradiated, including eight in each lung (Figure 1) at our hospital. We gave 40 Gy(relative biological effectiveness (RBE)) to a smaller lesion and 50 Gy(RBE) to other larger lesions in a single fraction. For the bone metastases, we gave CIRT 64 Gy(RBE) in 16 fractions to the right scapular lesion and 60 Gy(RBE) in 12 fractions to the ninth thoracic vertebra a week after lung irradiation.

Case 2 was an 81-year-old male patient with moderately differentiated adenocarcinoma of the ascending colon with multiple lung metastases on initial diagnosis, cT3N0M1. He had the comorbidity of old stroke and chronic obstructive pulmonary disease (COPD). He refused chemotherapy and had an upfront right hemicolectomy and lymph node dissection. The pathological stage was pT3N0M1. Physical examination was unremarkable. A pulmonary function test confirmed the diagnosis of grade 2 COPD. On February 27, 2023, the patient underwent CIRT for all 15 lesions of the left side lung, sparing the right lung. We gave 50 Gy(RBE) in one fraction. The lesion size was between 0.5 and 3.9 cm.

Carbon ion radiotherapy plan and HFOV

**Figure 2:**
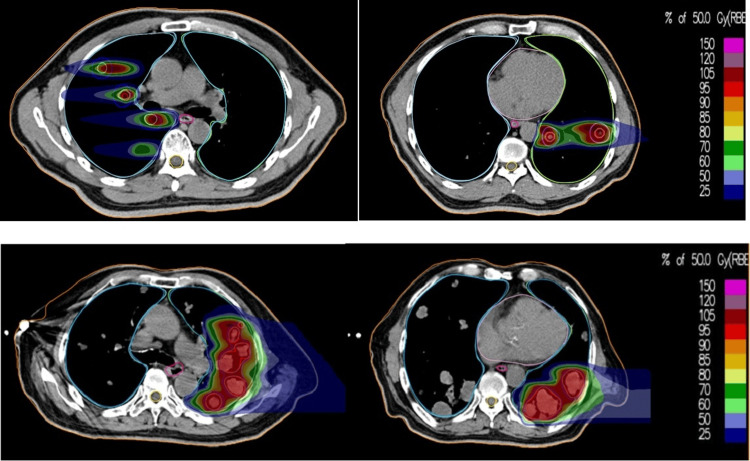
The dose distribution of carbon ion radiotherapy (CIRT) with a single dose of 50 Gy(RBE) The upper panel shows the dose distribution of Case 1, who had CIRT to bilateral lung lesions, showing a sharp dose fall-off. The V30, V20, V10, and V5 of total lung were 3.57%, 6.06%, 10.7%, and 14.95%, respectively. Because of the design of the treatment planning system, the right and left lung CIRT were planned separately. The lower panel shows the dose distribution of CIRT of Case 2, who had a left lung lesion CIRT, showing a sharp dose fall-off. The V30, V20, V10, and V5 of the total lung were 12.35%, 15.3%, 18.48%, and 21.05%. In an in silico run of photon radiotherapy planning, the V20 was 20% and V5 was 51.7% for total lung volume. RBE: relative biological effectiveness; V30: volume of the normal lung that receives 30 Gy or more carbon radiation; V20: volume of the normal lung that receives 30 Gy or more carbon radiation; V10: volume of the normal lung that receives 10 Gy or more carbon radiation; V5: volume of the normal lung that receives 5 Gy or more carbon radiation

The patients underwent four-dimensional (4D) CT simulation with free breathing and then with a high-frequency oscillatory (HFO) ventilator under general anesthesia. The latter set of CT images was imported into rtStation, a software of DaTu Medical Technology Co., Ltd. (Shanghai, China) for target delineation, which was then imported into the ciPlan CIRT planning system (ciPlan, Version 1.0, IMP, Lanzhou, China) for treatment planning. Gross target volume (GTV) was the lesion visible on chest CT under the lung window. The planning target volume (PTV) was GTV plus 3-5 mm. The targets of Case 1 are shown in Figure 1, and the isodose distributions of Cases 1 and 2 are shown in Figure 2. The assembly and setup of the HFO ventilator have been previously described in detail [13].

Both patients were irradiated under general anesthesia using a scanning beam of carbon ions delivered in one single fraction. Carbon ion was delivered by three non-coplanar ports with the couch at 0, +10, and -10 degrees for Case 1 and one port with the couch at 0 degrees for Case 2. Radiation dose-volume parameters including volume of the normal lung that receives 5 Gy or more carbon radiation (V5), V10, V20, V30, and mean lung dose were also derived.

Results

Case 1

**Figure 3:**
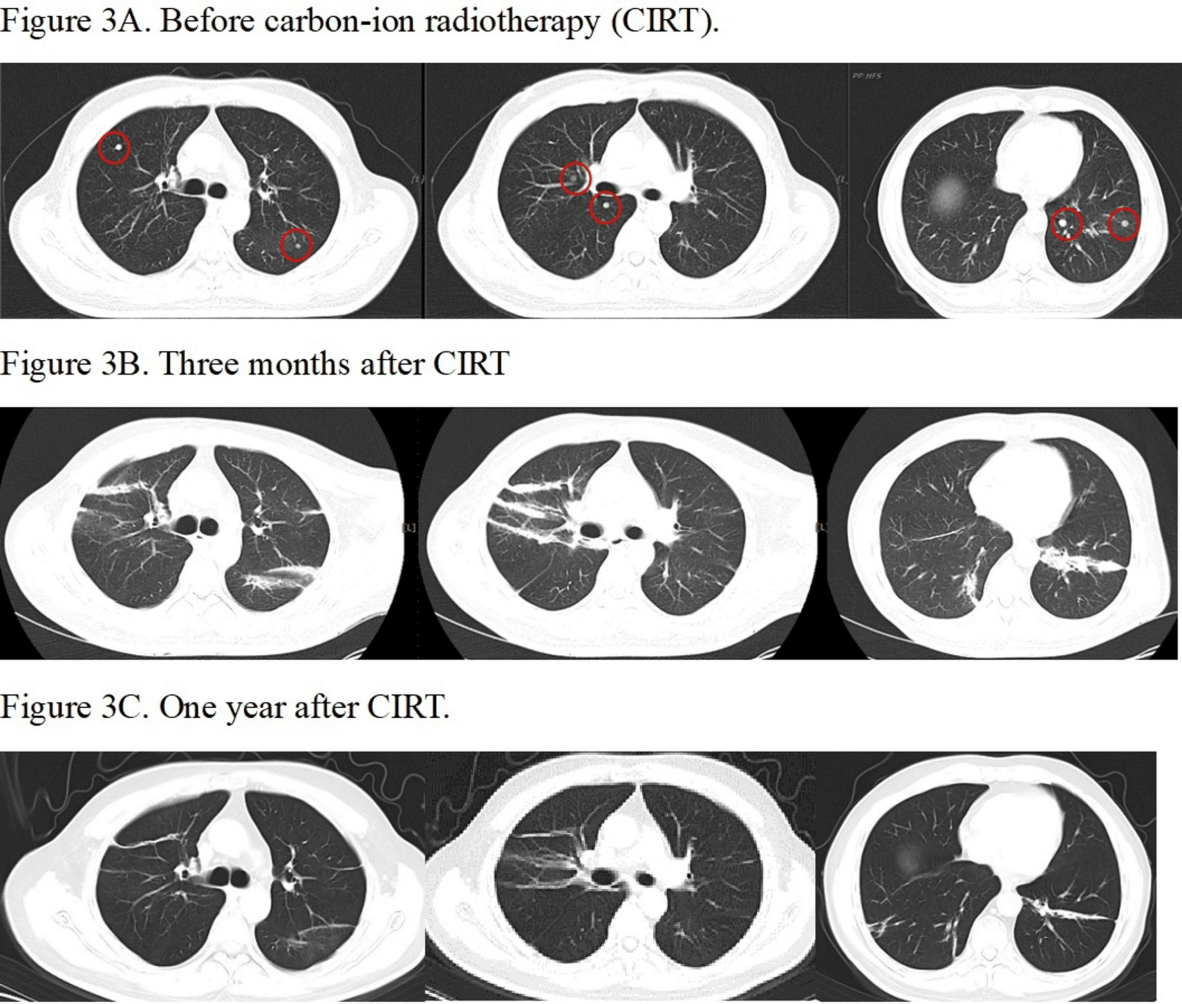
CT images of Case 1 before, three months, and one year after carbon ion radiotherapy (CIRT) to the bilateral lung Figure 3A demonstrates the CT images with lung window before CIRT. Eight lesions of each lung (red circles) were irradiated with a single dose of 50 Gy(RBE) (one small lesion near the heart had 40 Gy(RBE)). Figure 3B shows complete tumor regression three months after CIRT. There were radiopaque lesions along the path of carbon ions, representing radiation pneumonitis. Figure 3C shows linear tracts of lung fibrosis along the carbon ion path and complete regression of irradiated lesions one year post CIRT. By December 2024, 22 months after the initial total lung lesion CIRT, he has had another three CIRT courses for lung, liver, and bone progressions and is alive with good quality of life. RBE: relative biological effectiveness

The anesthesia time was 150 minutes. The maximum range of motion (MRM) of the diaphragm at free breathing versus HFOV with anesthesia was 1.15 cm versus 0.1 cm, respectively. The V30, V20, V10, and V5 of total lung were 3.57%, 6.06%, 10.7%, and 14.95%, respectively. After CIRT, the patient received pembrolizumab every three weeks. A CT of the chest showed a complete response three and 12 months after CIRT (Figure 3).

The patient had grade II pneumonitis with a mild cough, which resolved after oral prednisolone. However, new liver and bone lesions developed and were irradiated with 60 Gy(RBE) in four fractions of CIRT in Jan. 2024, 10 months after CIRT. By December 2024, he has had another three CIRT courses for lung, liver, and bone progressions. Presently, 22 months after CIRT, the irradiated lesions remained controlled, and the patient has good physical condition and no pulmonary complaint except asymptomatic lung fibrosis. He has discontinued pembrolizumab and shifted to lenvatinib 8 mg once a day (QD), and now he is receiving cadonilimab (a Chinese brand of dual PD-1 and CTLA-4 inhibitors) plus lenvatinib.

Case 2

**Figure 4:**
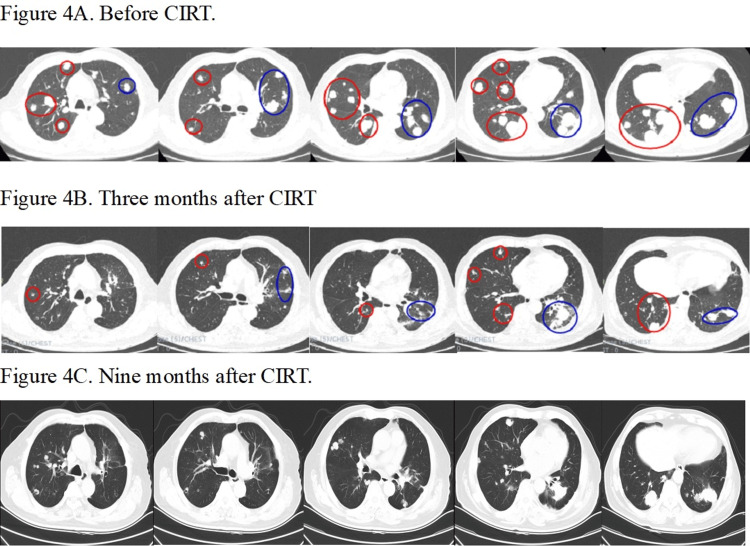
CT images of Case 2 before and after carbon ion radiotherapy (CIRT) Figure 4A demonstrates the CT images with the lung window before CIRT. A total of 15 lesions of the left lung were irradiated (blue circle) with a single dose of 50 Gy(RBE). The lesions of the right lung were not irradiated due to the concern of overdosing the normal lung. Figure 4B shows the partial tumor response (blue circle) of the irradiated lesions three months after CIRT. The unirradiated right lung lesions (red circle) also show a significant size reduction, demonstrating a possible abscopal effect (the patient started oral capecitabine two weeks after CIRT). There were no signs of radiation pneumonitis. Figure 4C reveals continuing tumor control at the irradiated left lung nine months after CIRT, but a few unirradiated lesions at the right lung become larger. By December 2024, 22 months after CIRT, he has had disease progression at the primary colon and right lung with elevated CEA and no cancer treatment. There is no radiation pneumonitis or lung fibrosis. He is under hospice care presently. RBE: relative biological effectiveness; CEA: carcinoembryonic antigen

The anesthesia time was 160 minutes. The MRM of the diaphragm at free breathing versus HFOV was 3.09 cm and 0.2 cm, respectively. The V30, V20, V10 and V5 of total lung was 12.35%, 15.3%, 18.48% and 21.05%, respectively. The treated lesions showed a partial response. The unirradiated right lung lesions also showed a significant reduction in size (Figure 4).

It is not certain whether this is an abscopal effect of CIRT or not because the patient started to take oral capecitabine daily two weeks after CIRT. His carcinoembryonic antigen (CEA) level dropped from 102 before CIRT to a nadir of 22.28 six months after CIRT. By December 2024, which was 22 months after CIRT, he had disease progression at the right lung with elevated CEA and no cancer treatment. There is no radiation pneumonitis or lung fibrosis. The patient is presently in hospice care.

## Discussion

Conventionally, for patients with multiple lung metastases, radiotherapy is purely for symptom relief with a lower radiation dose. However, the concept of oligometastases suggests that local treatment such as radiotherapy can prolong life and potentially cure the patient in such patients [14]. Clinically, this hypothesis has been confirmed by retrospective and phase II and III studies, which reported a survival benefit of metastasis-directed radiotherapy. Taking as an example, a retrospective analysis of 71 patients with mostly one to two lung metastases treated with a median of 60 Gy in eight fractions of stereotactic body radiotherapy (SBRT) reported a four-year local control of 90% [15]. The phase II Stereotactic Ablative Radiotherapy for the Comprehensive Treatment of Oligometastases (SABR-COMET) trial shows a five-year overall survival of 17.7% in patients treated with palliative radiotherapy versus 42.3% in patients treated with curative SABR to all metastatic lesions in the lung [16]. Currently, the concept of oligometastases is being evaluated for possible extension beyond the traditional one to five metastases. While the SABR-COMET trial included patients with a maximum of five lesions, the phase I Ablative Radiation Therapy to Restrain Everything Safely Treatable (ARREST) trial evaluated the safety and feasibility of SABR to all polymetastatic sites in patients with more than 10 metastases [17]. Thirteen patients were treated, with a median number of 16 metastases (range 11-27) per patient. The results show that with 6 Gy weekly and a total of 30 Gy in five fractions, SABR could be given safely. The ongoing phase III SABR-COMET-10 assesses the impact of SABR in patients with four to 10 metastatic cancer lesions [18]. Brooks and Chang propose that when combining radiotherapy and immune checkpoint inhibitors, the larger the irradiated volume, the better the radiation-induced immune response [19]. By giving definitive radiotherapy to all metastatic lesions of patients, such as our reported cases, there exists a potential additional benefit of a high degree of radiation-induced anti-cancer immune response.

Both of our patients have survived 22 months and are still alive. In a German study, the four-year overall survival was 39.7% in a patient population with mostly one to two metastatic lesions [15]. The SABR-COMET, which enrolled patients with one to five metastases, reported a median overall survival of 50 months [16]. These data seem to demonstrate that even with so many metastatic lesions, patients can still survive a considerable period of time after curative-dose, metastasis-directed radiotherapy.

For Case 1, the progression-free survival was 10 months. This compares favorably to the 3.6 months reported by the ARREST trial [17] and is comparable to the 11.6 months by SABR-COMET [16]. Case 1 has had another three CIRT courses after the initial one. Repeated irradiation was also conducted in 29.5% of patients in the retrospective study from Germany (15) and 38% of patients in the ARREST trial [17].

Regarding safety, one of our patients had grade II radiation pneumonitis that resolved after steroid administration. The same patient showed radiation fibrosis that was asymptomatic. The other patient had no radiation pneumonitis. A similar safety profile was reported by the above-mentioned publications, where SABR was generally well tolerated with a low risk of grade III or higher toxicity [15-17].

The ability to treat 15 or more lung lesions in one dose was based on three attributes of this technique. Firstly, high-dose carbon ions can be safely delivered without exceeding the dose constraints of the normal lung because of its physical characteristics of dose distribution. Secondly, HFOV was able to control the maximum respiration motion down to 1-2 mm during CIRT to allow for high-precision irradiation. Thirdly, HFOV under general anesthesia also made the procedure tolerable to treat a large number of lung lesions in one session of treatment.

Due to the high capital investment, CIRT is currently a costly therapy modality, approximately three to five times that of photon radiotherapy. Whether CIRT is worth the cost depends on the benefits that it confers to patients. In these two cases, CIRT was their last resort, and it seems to have prolonged their life span.

## Conclusions

Total tumor CIRT with HFOV provides a novel potential option in patients with multiple lung metastases who are unfit or refractory to systemic or local therapies and could contribute significantly to the multimodality management of patients with multiple lung metastases. However, further clinical studies in a larger patient population are needed before CIRT with HFOV could be recommended as a routine treatment modality for patients with more than 10 lung metastases.
